# Review Areca Thirteen Pill (GY-13) for depression: a ConPhyMP-Informed critical review of material definition, pharmacological evidence, and translational Heyi-neuroplasticity-stress hypotheses

**DOI:** 10.3389/fphar.2026.1912784

**Published:** 2026-09-18

**Authors:** Narisu Dai, Zhiying Bao, Xiaomin Chen

**Affiliations:** 1 Baotou Medical College, Inner Mongolia University of Science and Technology, Baotou, China; 2 Department of Mongolian Medicine, Inner Mongolia Minzu University, Tongliao, China

**Keywords:** Areca Thirteen Pill, BDNF, ConPhyMP, depression, four pillars of ethnopharmacology, GY-13, Heyi, HPA axis

## Abstract

Previous summaries of GY-13 have lacked transparent literature-search methods, reproducible material definitions, chemical-characterization status, or critical evidence grading, limiting their scientific utility.

**Objective:**

This review critically evaluates what the published GY-13 literature shows and does not show regarding antidepressant pharmacology, material reproducibility, clinical evidence, and safety. It also reformulates the Heyi-Neuroplasticity-Stress (HNS) framework as a testable translational hypothesis rather than as a confirmed mechanism.

**Methods:**

This critical scoping review was reported using a PRISMA-informed approach, with PRISMA-ScR, the Four Pillars of Best Practice in Ethnopharmacology, and ConPhyMP used as reporting and interpretive frameworks. Searches were rerun on 3 June 2026 in PubMed/MEDLINE and CNKI, supplemented by public bibliographic records and citation checking. Thirty records were identified; after deduplication and screening, 12 studies or contextual records were retained. The review was not prospectively registered, screening and extraction were not performed by two independent reviewers, and no validated design-specific risk-of-bias tool was applied. These limitations preclude designation as a fully systematic review.

**Results:**

The available evidence supports the possibility that GY-13 affects depression-related behavioral and molecular endpoints in stress-based animal models, including cAMP/PKA/CREB/BDNF signaling, HPA-axis feedback, monoamine signaling, inflammatory-oxidative pathways, and chemokine-axis findings. However, the reproducibility of these findings is limited by incomplete reporting of batch identity, voucher/authentication data, manufacturing details, HPLC/UPLC or LC-MS fingerprints, marker-metabolite quantification, dose-response structure, and randomization/blinding. Human evidence remains preliminary and insufficient for definitive clinical-efficacy claims.

**Conclusion:**

GY-13 is best treated as a hypothesis-generating multi-component intervention requiring chemically defined batches, strict ConPhyMP documentation, independent preclinical replication, and well-powered clinical trials with standardized safety monitoring. The HNS framework may guide future biomarker-enriched research only if tested prospectively with transparent materials and methods.

## 1 Introduction

Major depressive disorder is a heterogeneous syndrome involving mood, cognition, sleep, appetite, stress physiology, immune tone, and social functioning. Monoamine-centered treatment remains clinically important, but contemporary depression biology increasingly emphasizes interacting stress-neuroplasticity, endocrine, inflammatory, oxidative, and metabolic networks. This shift creates a rationale for evaluating multi-component botanical and traditional medicine interventions. Such evaluation is meaningful only when the tested materials and pharmacological evidence are defined well enough for replication (Duman and Monteggia, 2006; Pariante and Lightman, 2008; Pittenger and Duman, 2008; Felger and Lotrich, 2013; Miller and Raison, 2016).

Mongolian medicine interprets many depression-like presentations through Heyi-related pathology, especially Heyi excess, White Meridian obstruction, and imbalance among the three roots. Areca Thirteen Pill (GY-13, Gaoyou-13, Binlang Shisanwei Wan) is a representative Zhen Heyi formula historically used for psycho-somatic and heart-brain symptoms. Several experimental studies, especially from Tong and colleagues and related groups, have reported effects on depression-related behavior, cAMP/PKA/CREB/BDNF signaling, HPA-axis regulation, monoamines, and other molecular pathways (Tong et al., 2012; Bao et al., 2014; Tong et al., 2014a; Tong and Bai, 2014b; Cai et al., 2022; Bai et al., 2024; Li et al., 2025).

This review is structured as a PRISMA-informed critical scoping review rather than a fully systematic review or narrative summary. Its primary contribution is not to assert clinical efficacy for GY-13, but to construct an evidence map that systematically distinguishes reported pharmacological findings from unresolved gaps in material characterization, study design, and clinical validation. This approach is consistent with the methodological expectations for ethnopharmacological reviews that require transparent search protocols, reproducible material definitions, and critical evidence grading.

This review has five aims. First, it provides a transparent review method and data-extraction framework. Second, it defines GY-13 ingredients according to taxonomic and material-reporting standards. Third, it evaluates preclinical, computational, clinical, and safety evidence using evidence tiers. Fourth, it reframes HNS as a testable translational hypothesis. Fifth, it establishes priorities for reproducible pharmacological and clinical research.

## 2 Methods

### 2.1 Review design and reporting framework

This article is a PRISMA-informed critical scoping review and does not present new experimental chemistry, pharmacology, or clinical data. PRISMA 2020 and PRISMA-ScR informed reporting and study-flow presentation (Page et al., 2021; Tricco et al., 2018), while the Four Pillars and ConPhyMP guided appraisal of ethnopharmacological material definition and chemical characterization (Heinrich et al., 2020; Heinrich et al., 2022). The separate ConPhyMP supporting file contains the material/processing and chemical-profile tables.

No prospective protocol was registered. Study screening and data extraction were not performed independently by two reviewers, and no validated, design-specific risk-of-bias instrument was applied. Consequently, this article should not be interpreted as a fully systematic review or a formal risk-of-bias assessment. Instead, it maps the accessible evidence and treats missing information as an evidentiary result: absent batch, voucher, chemical fingerprint, dose, model, or clinical details directly reduce confidence in reproducibility and inference. These limitations are revisited in Section 9.2.

### 2.2 Search strategy

The database rerun was conducted on 3 June 2026. The primary database search covered PubMed/MEDLINE and CNKI. It was supplemented by accessible Chinese and English bibliographic pages and backward/forward citation checking. Web of Science and Scopus were not searched as independent primary databases and have therefore been removed from the database-count table. The reproducible search strings and the role of each source are shown in Table 1. The public rerun identified 30 records: PubMed/MEDLINE, n = 3 directly relevant English biomedical records; CNKI/public Chinese records, n = 8; citation/reference and guideline searches, n = 19. After removing five duplicates or duplicate notices, 25 records were screened. Ten records were excluded at title/abstract/public-record screening because they were not GY-13-specific, not depression-related, or used only as general background. Fifteen full-text articles or detailed public records were assessed; three were excluded from evidence extraction because they were not primary GY-13 evidence, lacked usable material or outcome data, or duplicated method context. Twelve studies/records were retained for critical extraction: five preclinical records involving *in vitro* or animal pharmacology, two computational records involving network pharmacology or other *in silico* analyses, two clinical or clinical-observation records, and three material/safety/context records. No publication-date restriction was applied; English- and Chinese-language records were eligible.

**TABLE 1:** Search sources, reproducible search strings, and scope.

Database	Search string	Purpose
PubMed/MEDLINE	(“Areca Thirteen Pill” OR GY-13 OR Gaoyou-13 OR “Binlang Shisanwei” OR “Arecae semen thirteen pill”) AND (depression OR depressive OR antidepressant OR “major depressive disorder” OR CUMS OR “chronic unpredictable mild stress”)	English biomedical and pharmacological records; public rerun 3 June 2026 identified 3 directly relevant PubMed records
CNKI	(槟榔十三味丸 OR 高尤十三味丸 OR GY-13 OR 蒙药槟榔十三味丸) AND (抑郁 OR 抗抑郁 OR 慢性应激 OR HPA OR cAMP OR PKA OR CREB OR BDNF OR 临床)	Chinese primary studies and clinical reports; the rerun identified 8 relevant Chinese records or public bibliographic records for screening
Supplementary public records and citation chaining	Formula names above combined with depression, pharmacology, chemistry, or safety terms; reference lists of included studies and relevant guidelines were checked	Located contextual material, safety, and guideline records; these records were not treated as an independently comprehensive database search

Duplicate records were identified using combinations of title, first author, publication year, journal, and DOI or other bibliographic identifiers. Selection then proceeded in two stages. First, titles and abstracts or public-record summaries were screened against the intervention, condition, and study-type criteria in Table 2. Second, potentially eligible full texts or detailed public records were assessed for a recognizable GY-13 intervention and usable material, model or population, and outcome information. Exclusion reasons were recorded at the applicable stage and are summarized in Figure 1.

**TABLE 2:** Eligibility criteria.

Domain	Inclusion criteria	Exclusion criteria
Study type	Traditional/textual formula studies, material and quality-control studies, *in silico*/network pharmacology, *in vitro* pharmacology, animal pharmacology, clinical studies, real-world evidence, and safety studies relevant to GY-13 or its major safety risks	General depression reviews without GY-13, formula-irrelevant papers, commentary without usable evidence, duplicate datasets, and records without retrievable methodological details unless cited only to show evidence gaps
Intervention/material	GY-13, Gaoyou-13, Binlang Shisanwei Wan, Areca Thirteen Pill, or directly relevant ingredients/safety markers	Undefined multi-herb mixtures not corresponding to GY-13, unless used only as comparator context
Pharmacological data	Studies reporting at least the model, intervention, endpoint, and evidence direction	Studies making only broad activity claims without usable information on model, dose, endpoint, or material
Clinical data	Human studies reporting depression, depressive symptoms, anxiety/depression comorbidity, or related endpoints with GY-13 or closely related Zhen Heyi formulas	Clinical anecdotes that cannot identify population, intervention, comparator, outcome, or safety monitoring

**FIGURE 1:**
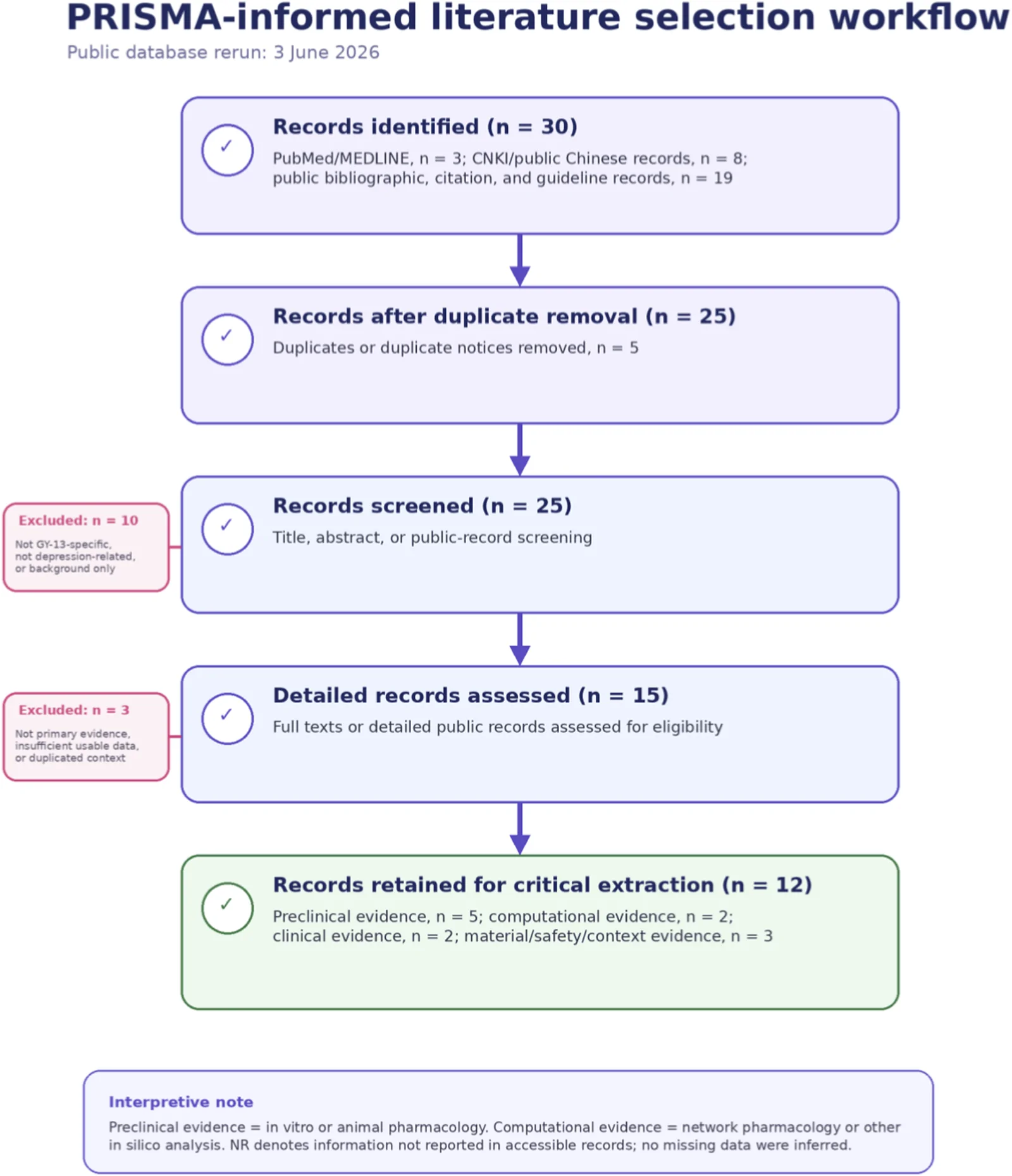
PRISMA-informed literature selection workflow for the present review. Counts reflect the documented public database rerun performed on 3 June 2026. The figure distinguishes records retained for evidence extraction from records used only as background or methodological context.

### 2.3 Eligibility criteria

Eligibility criteria.

### 2.4 Data extraction and quality domains

For pharmacological studies, extraction covered the test material, supplier, manufacturer, lot number, voucher or authentication record, formulation ratio, and preparation method. It also covered dose or concentration, route, duration, species, strain, sex, model, controls, randomization, blinding, sample size, endpoints, statistics, adverse events, and links between chemistry and bioactivity. For clinical studies, extraction covered diagnostic criteria, sample size per arm, randomization, allocation concealment, blinding, intervention and comparator, depression scales, numerical outcomes, adverse events, follow-up, funding, and conflicts of interest. The documented review process did not include duplicate, independent extraction; this is acknowledged as a limitation. No missing value was imputed. “NR” denotes information not reported in the accessible full text or public record.

### 2.5 Critical appraisal and evidence-tier assignment

Because the included evidence spans textual sources, network pharmacology, cell experiments, animal studies, and limited clinical observations, a single validated appraisal instrument would not cover all designs. A *post hoc* formal SYRCLE, RoB 2, or ROBINS-I assessment was not undertaken. Instead, each record was descriptively appraised across prespecified domains: material identity and chemical definition; model or population; dose and exposure; comparator; sample size; randomization; blinding; endpoint validity; statistical reporting; adverse-event reporting; and independence of replication. This domain review supports interpretation but is not presented as a validated risk-of-bias score.

Evidence tiers were assigned by study design, not by direction or statistical significance of results. When one publication included several designs, each component was interpreted at its own tier. Missing reporting did not automatically change the design tier, but it reduced confidence within that tier. Tier five was reserved for adequately powered randomized clinical trials; none was identified.

Within each tier, the strength of support for a specific claim was judged qualitatively. Stronger support required a defined test material, an appropriate model or population, explicit dose and exposure, suitable controls, an adequate sample size, randomization and blinded outcome assessment where applicable, validated endpoints, complete statistical reporting, safety reporting, and independent replication. Missing information in several domains resulted in limited or very limited confidence. No included GY-13 study provided high-confidence clinical-efficacy evidence Table 3.

**TABLE 3:** Operational evidence-tier rubric used in this review.

Tier	Evidence type	Permitted inference in this review
0	Traditional/textual rationale	Supports cultural and therapeutic context only; does not establish pharmacological efficacy
1	Network pharmacology/*in silico* prediction	Generates hypotheses about metabolites, targets, and pathways; does not prove exposure, target engagement, or activity
2	*In vitro* pharmacology	Supports mechanistic plausibility only if concentration, material, and controls are defined
3	Animal pharmacology	Supports preclinical plausibility if dose, model, controls, randomization, blinding, and material definition are adequate
4	Observational or real-world clinical evidence	Suggests clinical signal but is vulnerable to confounding and indication bias
5	Randomized controlled clinical trial	Can support clinical efficacy only if adequately powered, transparently reported, and supported by safety data. Current GY-13 evidence does not yet reach this standard

### 2.6 Synthesis

Given the heterogeneity of study designs, outcomes, and incomplete numerical reporting, meta-analysis was neither planned nor appropriate. Findings were synthesized narratively by evidence tier and biological domain. In this review, “preclinical evidence” refers only to *in vitro* or animal pharmacology. “Computational evidence” refers to network pharmacology and other *in silico* analyses and is treated as hypothesis-generating. “Mechanistic evidence” is reserved for experiments that directly test causality, such as inhibitor, knockdown, or target-engagement studies. Ingredient-level safety evidence was not used to quantify formula-level risk.

## 3 Results

The results begin here. They are organized as study selection and evidence landscape (Section 3.1), formula identity and material reproducibility (Sections 3.2–3.4), traditional-theory context (Section 4), pharmacological evidence (Section 5), and clinical/safety evidence (Section 6). Figure 1 reports the selection flow; tables summarize rather than replace the narrative.

### 3.1 Study selection and evidence landscape

Of 30 identified records, five duplicates or duplicate notices were removed and 25 records were screened. Fifteen records underwent full-text or detailed public-record assessment, and 12 were retained for critical extraction. These comprised five preclinical records involving *in vitro* or animal pharmacology, two computational records involving network pharmacology or other *in silico* analyses, two clinical or clinical-observation records, and three material/safety/context records. The small number, mixed designs, and limited reporting precluded quantitative pooling. The evidence base is therefore an evidence map, not an efficacy estimate.

### 3.2 Formula identity and ingredient verification

GY-13 is a complex Mongolian formula containing botanical materials and a mineral/inorganic ingredient. Under the Four Pillars and ConPhyMP expectations, listing only Latin binomials is insufficient. Each ingredient should be reported with accepted scientific name, family, plant part or pharmacopoeial drug name, source, processing, and quality-control status. The following matrix is written in a form suitable for the manuscript, while final accepted names and pharmacopoeial drug names should be checked against Plants of the World Online, MPNS, the Chinese Pharmacopoeia, and relevant Mongolian medicine standards. The names below reproduce the formula identity reported in the included textual/formula sources, especially Tong and Bai, (2014b); they are not new ingredients proposed by the authors. Unresolved synonyms and drug-part details are explicitly labeled “to verify.” Thus, the table distinguishes the reported formulation from author-side nomenclatural normalization Table 4.

**TABLE 4:** Reported GY-13 ingredients and material-definition requirements.

Ingredient reported for GY-13	Material definition to use/verify	Family or material class	Plant part/drug name to verify	Critical reproducibility or safety note
Areca catechu L	Areca catechu L	Arecaceae	Arecae semen/seed	Central safety concern: arecoline and related alkaloids; oral mucosal, carcinogenicity, cholinergic and dependence risks must be discussed
Aquilaria agallocha Roxb	Aquilaria spp.; accepted name requires verification	Thymelaeaceae	Aquilariae lignum resinatum/resinous wood	High substitution/adulteration risk; source and authentication needed
Myristica fragrans Houtt	Myristica fragrans Houtt	Myristicaceae	Myristicae semen/seed	CNS-active metabolites at high exposure; dose and safety data should be reported
Syzygium aromaticum (L.) Merr. & L.M.Perry	Syzygium aromaticum (L.) Merr. & L.M.Perry	Myrtaceae	Caryophylli Flos/flower bud	Eugenol is a plausible marker metabolite, but GY-13 batch content is not established
Aucklandia lappa decne	Aucklandia costus Falc./Aucklandia lappa decne.; accepted name to verify	Asteraceae	Aucklandiae radix/root	Costunolide and dehydrocostus lactone are plausible marker metabolites
Choerospondias axillaris (Roxb.) B.L.Burtt & A.W.Hill	Choerospondias axillaris (Roxb.) B.L.Burtt & A.W.Hill	Anacardiaceae	Fruit/Choerospondiatis Fructus to verify	Plant part and source must be standardized
Aconitum brachypodum diels	Aconitum brachypodum diels	Ranunculaceae	Processed aconitum material to verify	High-risk ingredient; processing, toxic alkaloids, and detoxification controls must be specified
Zingiber officinale Roscoe	Zingiber officinale Roscoe	Zingiberaceae	Zingiberis rhizoma/rhizome	Gingerols/shogaols are plausible marker metabolites
Piper longum L	Piper longum L	Piperaceae	Piperis Longi fructus/fruit	Piperine-related pharmacokinetic interaction risk
Piper nigrum L	Piper nigrum L	Piperaceae	Piperis fructus/fruit	Piperine-related pharmacokinetic interaction risk
Sal ammoniac	Ammonium chloride/Sal ammoniac	Mineral/inorganic salt	Mineral ingredient	Requires purity, dose, contaminant, and pharmacopoeial quality-control information
Angelica sinensis (Oliv.) diels	Angelica sinensis (Oliv.) diels	Apiaceae	Angelicae sinensis radix/root	Ligustilide-related phthalides are plausible marker metabolites
Lepidium apetalum willd	Lepidium apetalum willd	Brassicaceae	Lepidii semen/seed to verify	Seed identity, source, and pharmacopoeial status must be verified

### 3.3 Material and initial-processing reproducibility

The material-level assessment asks whether the GY-13 batches used in pharmacological studies can be reproduced. Accessible reports occasionally provide a supplier, voucher, or dose, but most do not provide a complete chain from authenticated ingredients to a manufacturing lot. Detailed extraction is supplied in Supplementary Data Sheet 1, Table 1; the principal findings are summarized below.

The complete item-by-item table is provided in Supplementary Data Sheet 1; no duplicate table is retained in the main text.

### 3.4 Chemical-characterization status

This section evaluates whether measured chemistry is linked to the material used in bioactivity studies. Database-derived candidate compounds are not equivalent to measured constituents of an administered batch. No accessible record established a validated, batch-resolved chemical fingerprint linked to antidepressant outcomes. Detailed items are reported in Supplementary Data Sheet 1, Table 2a.

## 4 Mongolian medicine rationale and Heyi theory

This section provides the traditional-theory context needed to interpret why GY-13 has been studied for depression; it does not constitute efficacy evidence and is assigned Tier 0. Mongolian medicine is organized around the dynamic balance of Heyi, Xila, and Badagan. Heyi is associated with movement, respiration, circulation, sensation, mental activity, and regulatory mobility Figure 2. Depression-like presentations are commonly interpreted through Heyi excess, disturbance of heart-brain function, and progression toward White Meridian obstruction Table 5. Symptoms such as insomnia, palpitations, anxiety, dream disturbance, sadness, cognitive slowing, and reduced responsiveness can be interpreted within this theoretical frame. However, for a pharmacological review, this traditional rationale should be treated as a source of testable hypotheses rather than as evidence of molecular efficacy (Bai et al., 2014; Chen et al., 2019; Li et al., 2016).

**FIGURE 2:**
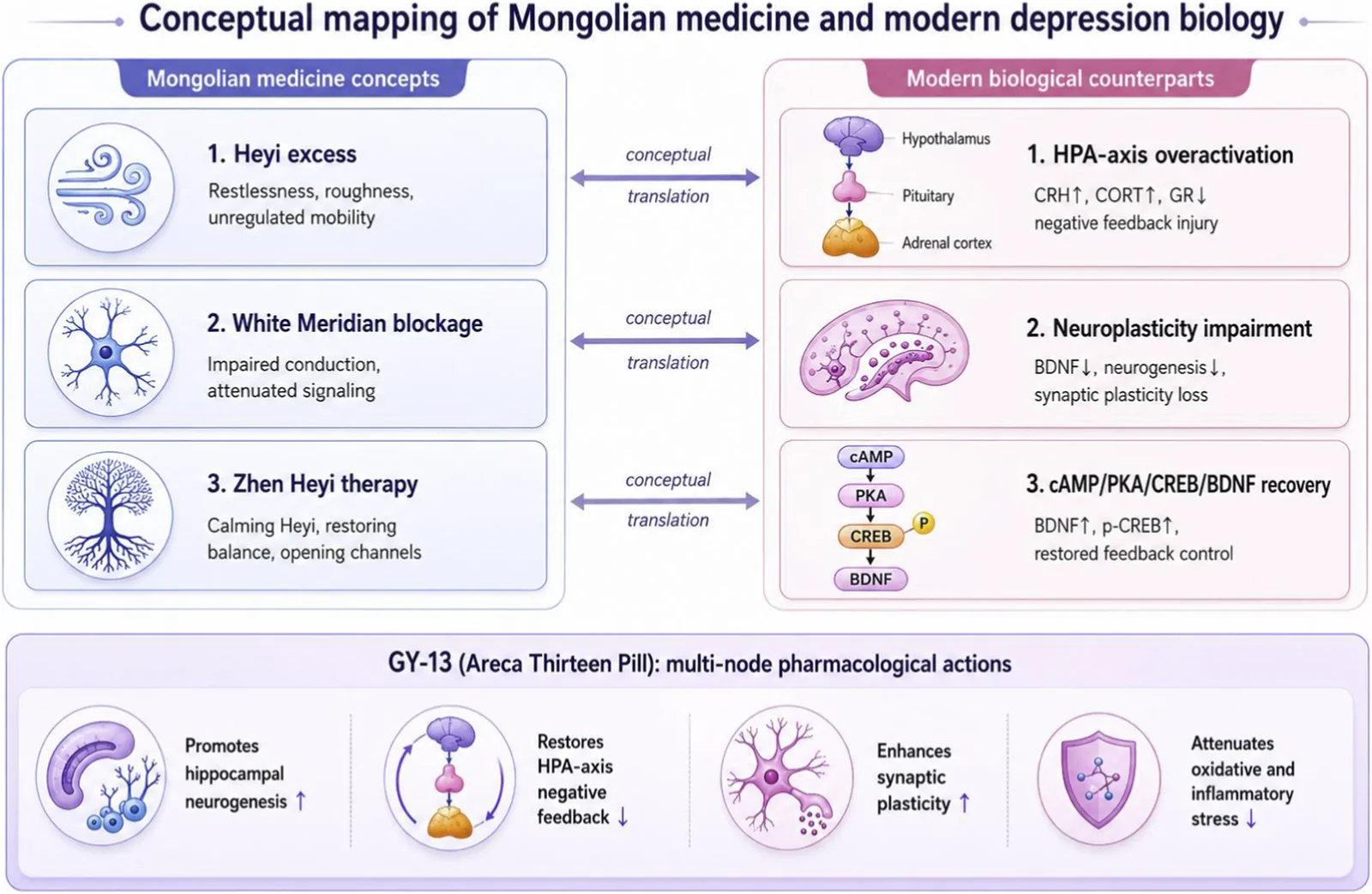
Conceptual mapping between Mongolian medicine Heyi theory and modern depression biology. In this review, this mapping is treated as a heuristic rather than evidence of equivalence.

**TABLE 5:** Heyi-related constructs, working hypotheses, and measurable variables.

Heyi-related construct	Traditional clinical meaning	Biomedical working hypothesis	Measurable variables
Heyi excess	Excessive mobility, restlessness, fear, insomnia, palpitations	Stress-axis overactivation and impaired negative feedback	Cortisol rhythm, CRH, ACTH, GR expression/function, sleep and autonomic measures
White Meridian obstruction	Impaired neural conduction, slowed thinking, memory decline, reduced responsiveness	Reduced neuroplasticity reserve	BDNF, TrkB, p-CREB, synaptic proteins, neurogenesis markers, cognitive/psychomotor tests
Three-root imbalance	Loss of systemic coordination among mental, somatic, digestive, and circulatory functions	Neuro-endocrine-immune dysregulation	Inflammatory markers, oxidative stress, metabolomics, symptom clusters
Zhen Heyi therapy	Calming Heyi and restoring regulated function	Multi-domain restoration of stress feedback and plasticity	HPA load, BDNF-TrkB signaling, behavior, safety outcomes

## 5 Critical assessment of pharmacological evidence

This section reports the principal pharmacological findings and limitations before directing readers to the comparative table. The following assessment evaluates each accessible GY-13 study individually against predefined criteria derived from the ConPhyMP and Four Pillars frameworks. For each record, we extract and report, where available: material identity, model system, dose, route, duration, controls, randomization, blinding, sample size, endpoints, statistics, and adverse events. Broad statements about pathway regulation are not accepted as evidence unless accompanied by the specific experimental context that supports them. Across included studies, direct efficacy evidence was preclinical (Tier 2 or 3); network pharmacology was Tier 1 and did not elevate the certainty of animal findings Table 6.

**TABLE 6:** Critical evidence map of GY-13 pharmacological studies.

Study/design	Material, model, and exposure	Principal findings	Critical limitations
Cai et al. (2022); animal and neuronal pharmacology	GY-13 in a CUMS rat model and glutamate-stressed primary hippocampal neurons; supplier and voucher BUCM4111403 reported; fluoxetine and H-89 used	Behavioral and neuronal findings were associated with cAMP/PKA/CREB/BDNF signaling	Animal dose schedule, randomization/blinding, batch fingerprint, marker ranges, and complete safety reporting were unavailable in the accessible record; not clinical evidence
Tong et al. (2014b); animal study	GY-13 in a rat depression model; hippocampal and prefrontal AC-cAMP-PKA endpoints	Supports pathway-level plausibility	Dose, route, duration, controls, batch identity, randomization, and blinding were NR in the accessible summary
Tong et al. (2012) and Bao et al. (2014); animal studies	Chronic-stress depression models treated with GY-13	Reported behavioral and HPA-axis/GR-related changes	Sample-size justification, randomization/blinding, detailed dose, and batch chemistry were incompletely reported or NR.
Tong et al. (2014a); animal study	Depression-model rats; brain-region monoamine endpoints	Reported changes in 5-HT, norepinephrine, dopamine, or related markers	Monoamine changes do not establish antidepressant efficacy; material and design reporting were incomplete
Li et al. (2025); integrated pharmacology plus animal validation	Network/docking/MR/MD analyses; reported oral GY-13 doses of 0.15, 0.3, and 0.6 g/kg for 28 days	Prioritized SLC6A4 and candidate compounds, with experimental support for selected endpoints	Predicted compounds were not established as measured, bioavailable constituents of the administered batch; no batch-resolved fingerprint
Bai et al. (2024); proteomics/network pharmacology animal study	Male SD rats exposed to CUMS for 42 days; control, model, three GY-13 groups, and fluoxetine	Behavioral, histological, inflammatory, proteomic, and chemokine-axis findings support a neuroinflammatory hypothesis	Animal evidence only; dose values were NR in the accessible record, and batch chemistry and clinical relevance remain unresolved
Ingredient-level safety literature	Areca, aconitum, piper, and mineral-related evidence; not formula-batch-specific	Identifies safety signals requiring monitoring and chemical control	Cannot quantify GY-13 risk without formula-level dose, processing, exposure, and batch chemistry

### 5.1 What the evidence supports

The available evidence supports a limited conclusion: GY-13 has been reported to affect behavioral and molecular endpoints in preclinical depression-related models involving cAMP/PKA/CREB/BDNF signaling, HPA-axis feedback, monoamine signaling, inflammatory-oxidative pathways, and related stress biology. These findings justify further investigation and provide a rationale for testing HNS-related biomarkers (Tong et al., 2012; Bao et al., 2014; Tong and Wang, 2014a; Tong and Bao, 2014c; Cai et al., 2022; Bai et al., 2024; Li et al., 2025).

### 5.2 What the evidence does not support

The current evidence does not support definitive clinical efficacy, routine clinical recommendation, batch-to-batch reproducibility, a validated chemical quality-control specification, or a single confirmed mechanism of action. Computational studies do not demonstrate that predicted metabolites exist in active batches at bioavailable concentrations Figure 3. Animal findings do not establish human efficacy. Pathway changes should be described as associations unless causal involvement is directly tested through target engagement, inhibition or knockdown, pharmacokinetics, and independent replication.

**FIGURE 3:**
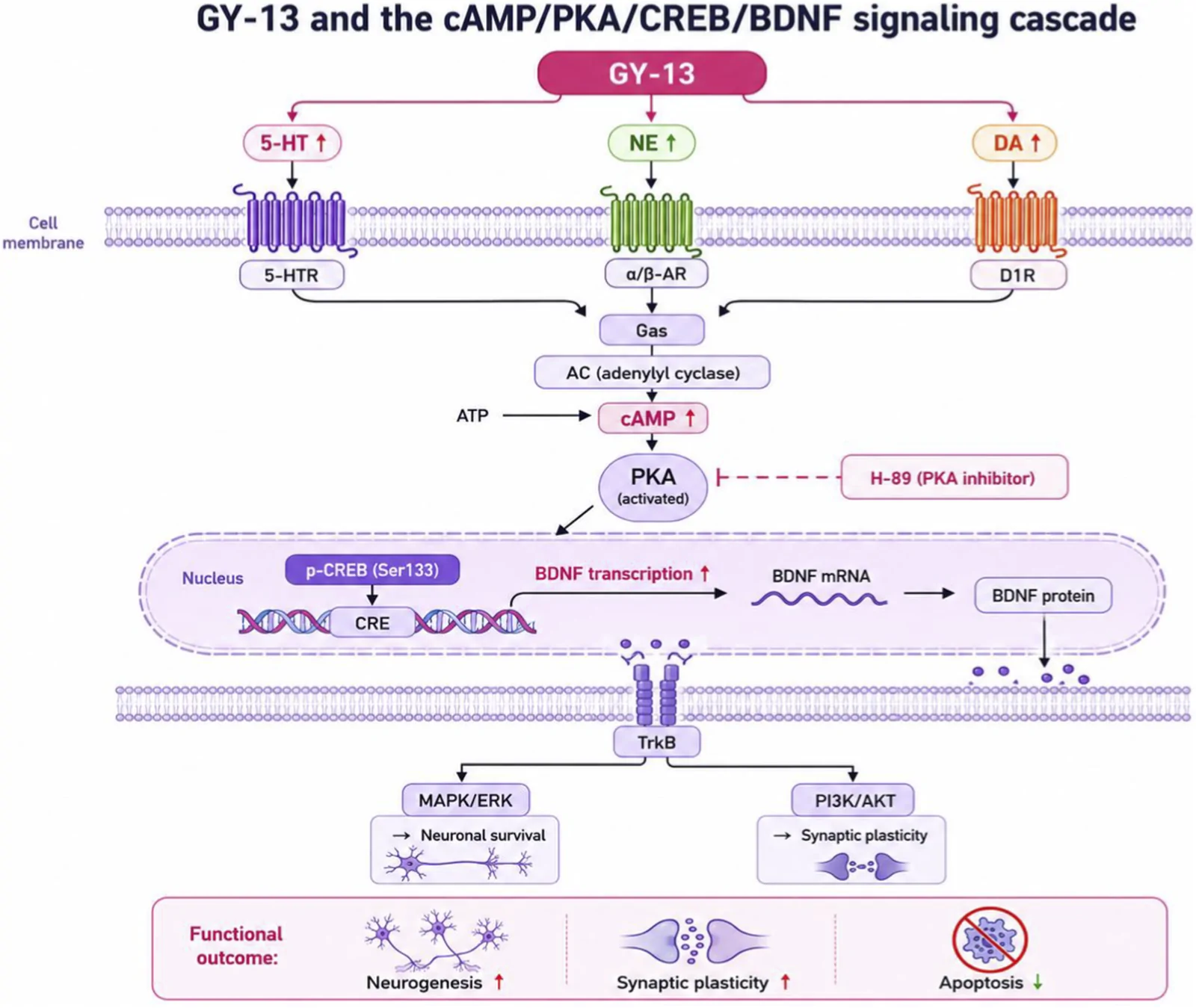
Preclinical cAMP/PKA/CREB/BDNF pathway hypothesis. Selected animal and neuronal studies report pathway-related associations; the diagram does not establish a clinical mechanism.

## 6 Clinical evidence and safety

This section separates human evidence from preclinical findings because only human studies can address clinical effectiveness and tolerability. Clinical evidence remains the weakest part of the current GY-13 depression literature. The Tai et al. (2025) public CNKI record describes a retrospective clinical comparison of 60 patients with mild-to-moderate depression treated from September 2023 to August 2024: 30 received sertraline-based treatment and 30 received GY-13 plus sertraline for 6 weeks. Outcomes included total clinical response, HAMD-17, MADRS, SF-36, PSQI, and adverse events. The public record reports a higher total effective rate in the combination group than in the sertraline group (93.33% vs 70.00%; chi-square = 5.455, P = 0.020), lower post-treatment HAMD-17 and MADRS scores in the combination group than in the control group (P < 0.01), and no significant difference in total adverse-event incidence (30.00% vs 26.67%; chi-square = 0.082, P = 0.775). However, the total sample was small (n = 60), the design was retrospective and non-randomized, and blinding was not reported Table 7. Exact baseline and endpoint scale values and full adverse-event categories were unavailable in the public record. The study is therefore treated as preliminary supportive evidence, not as definitive efficacy evidence (Tai et al., 2025).

**TABLE 7:** Clinical and translational evidence relevant to GY-13.

Clinical or translational evidence	Design/population	Intervention/comparator	Outcomes	Critical assessment
Early GY-13 clinical observation	Depressive symptoms; design details incomplete in accessible summaries	GY-13/Areca Thirteen Pill; comparator unclear or weakly controlled	Improvement in depressive symptoms reported	Clinical signal only; insufficient for efficacy claim without diagnostic criteria, sample size, comparator, validated scale, and safety monitoring
Tai et al., 2025 clinical report	Retrospective clinical comparison, 60 patients with mild-to-moderate depression treated from September 2023 to August 2024 at the Third People’s Hospital of Hulunbuir city; study group n = 30 and control group n = 30	Control: sertraline-based treatment. Study group: Mongolian medicine Binlang Shisanwei Pill/GY-13 combined with sertraline. Both groups were treated for 6 weeks	Total effective rate 93.33% vs 70.00% (chi-square = 5.455, P = 0.020). HAMD-17 and MADRS decreased after treatment in both groups and were lower in the combination group after 6 weeks (P < 0.01). SF-36 increased and PSQI decreased, with greater change in the combination group (P < 0.01). Total adverse-event incidence did not differ significantly (30.00% vs 26.67%; chi-square = 0.082, P = 0.775)	Clinical signal only. The public record supports sample size, treatment duration, broad outcomes and P values, but exact scale values, diagnostic criteria details, allocation concealment, blinding, adverse-event categories, and follow-up beyond 6 weeks remain unavailable from the public record. The study is retrospective and cannot establish definitive efficacy
Anshen Buxin Liuwei Pill/related real-world evidence	Hospitalized or post-PCI anxiety/depression contexts; not GY-13-specific	Related Mongolian Zhen Heyi formula	Signals of clinical utility in comorbid settings	Contextual evidence only; confounding and indication bias are likely; cannot be transferred directly to GY-13

### 6.1 Safety considerations

Safety cannot be treated as a secondary issue. Throughout this review, “adverse events” denotes unfavorable clinical occurrences reported during treatment, without presuming causality. “Adverse drug reactions” should be used only when a causal relationship is judged at least reasonably possible. “Herb–drug interaction” denotes a potential pharmacokinetic or pharmacodynamic interaction between GY-13 or its constituents and a conventional medicine.

GY-13 contains *Areca catechu*. Areca alkaloids such as arecoline raise concerns about oral mucosal injury, oral submucous fibrosis, oral-cancer risk, cholinergic adverse effects such as sweating, palpitation, and diarrhea, and dependence-related effects. English safety literature identifies areca-nut chewing as a major oral-cancer risk factor and summarizes large-magnitude human risks; mechanistic reviews also link arecoline and areca-nut constituents to oral submucous fibrosis and malignant transformation risk (Warnakulasuriya and Chen, 2022; Ekanayaka and Tilakaratne, 2016). The formula also includes Aconitum material, which requires verified processing and alkaloid control because Aconitum toxicity and cardiotoxicity are strongly dependent on processing, dose, and alkaloid profile (Zhang et al., 2022). Piper species may influence drug metabolism. Sal ammoniac and other mineral material require purity and contaminant testing. Therefore, future GY-13 clinical studies must include oral mucosal examination, liver and renal function, cardiovascular and cholinergic symptoms, neuropsychiatric adverse events, heavy metals and contaminants, and herb–drug interaction monitoring, especially if combined with SSRIs.

A definitive clinical-efficacy claim for GY-13 cannot be made until large, well-powered, independently conducted randomized controlled trials are completed using chemically defined batches and systematic adverse-event reporting. This review therefore does not recommend routine clinical use; it identifies the data required before such a recommendation would be scientifically defensible.

## 7 Heyi-neuroplasticity-stress framework as a testable hypothesis

The HNS framework is presented solely as a hypothesis-generating model. It does not claim that Heyi is identical to cortisol, that White Meridian obstruction is identical to BDNF loss, or that any proposed relationship is mechanistically confirmed. It identifies measurable interfaces between traditional clinical constructs and modern pharmacological endpoints. Future studies may test these associations prospectively by measuring stress-axis load, neuroplasticity reserve, and inflammatory-oxidative burden before and after GY-13 treatment Table 8.

**TABLE 8:** Operational HNS constructs and minimum validation requirements.

HNS construct	Operational biomarker domain	Minimum future experiment	Clinical-use hypothesis
Heyi excess/stress-axis load	Cortisol rhythm, CRH, ACTH, GR expression or function	CUMS model with dose-response, positive and negative controls, and GR-related endpoints; clinical cortisol rhythm before/after treatment	Patients with insomnia, palpitations, somatic arousal, anxiety, or high stress-axis markers may form a testable subgroup
White Meridian obstruction/neuroplasticity reserve	BDNF, TrkB, p-CREB, synaptic proteins, dendritic markers, cognitive/psychomotor endpoints	Defined GY-13 batch in animal and neuronal systems with inhibitors/knockdown and pre-specified endpoints	Patients with cognitive slowing, psychomotor retardation or low plasticity biomarkers may require neuroplasticity-focused endpoints
Inflammatory-oxidative burden	IL-1beta, TNF-alpha, CRP, ROS, antioxidant enzymes, metabolomics	Independent replication with inflammatory/oxidative stress assays and chemistry-linked dose-response	Patients with metabolic, inflammatory, cardiovascular, or somatic comorbidity may require enriched monitoring
Integrated response	Composite HPA-BDNF-inflammatory index with validated depression scales	Multi-omics linked to batch chemistry and behavior; computational model validated against data	Personalized trials can combine Mongolian syndrome differentiation with measurable biomarkers

The earlier HNS cycle and integrated future-strategy diagrams were moved to Supplementary Data Sheet 2 to reduce figure density and avoid duplicating the operational content in Figure 4 and Table 9.

**FIGURE 4:**
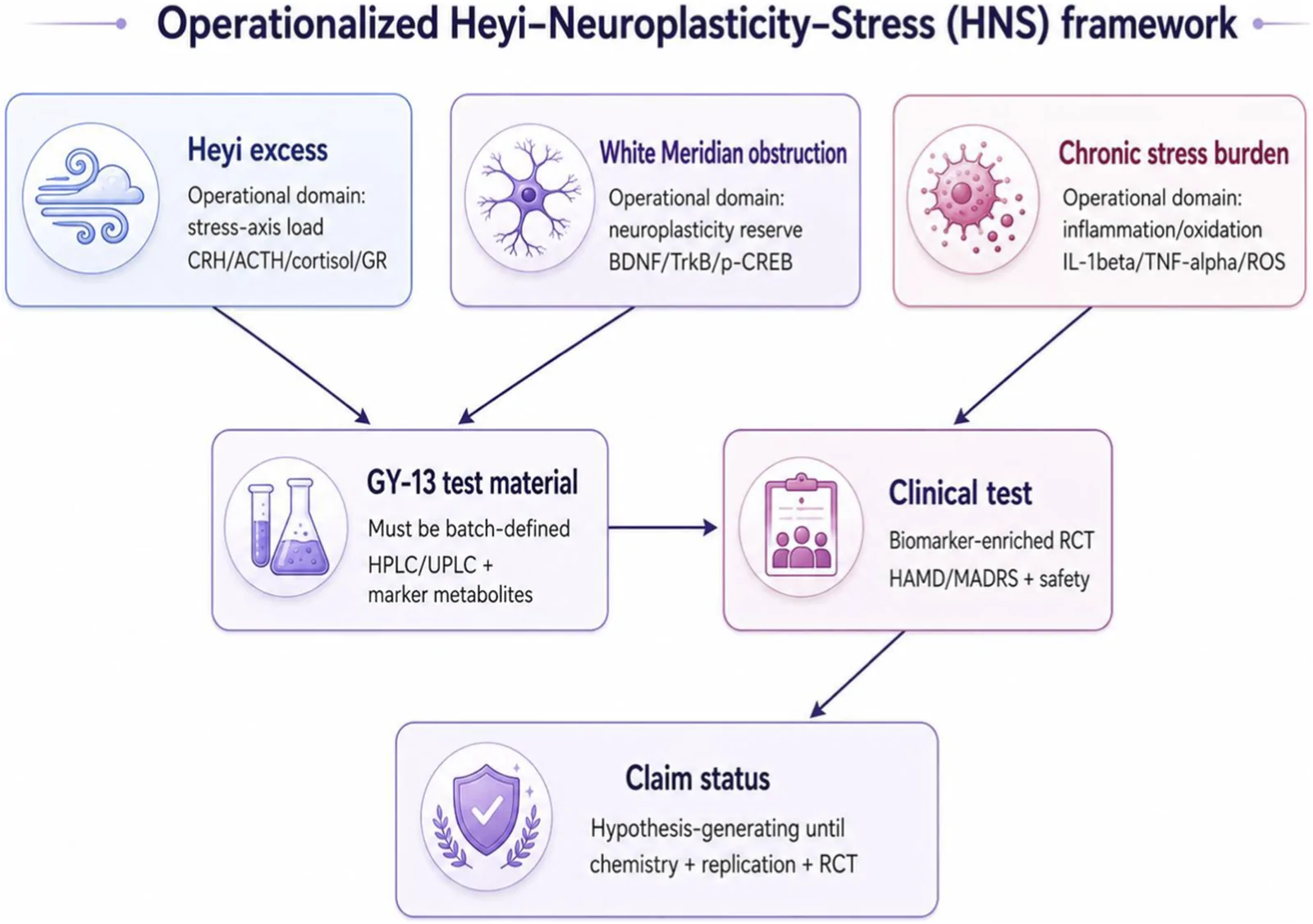
Operationalized HNS framework. Each traditional construct is linked to measurable biomarker domains and proposed validation studies. HNS is a hypothesis-development framework and does not claim mechanistic equivalence or confirmation.

**TABLE 9:** Prioritized research agenda for GY-13.

Priority	Requirement	Reason	Minimum deliverable
P0	Batch-defined GY-13 material	Without material definition, pharmacology cannot be repeated	Manufacturer, lot, formula ratio, processing, storage, voucher/authentication, HPLC/UPLC or LC-MS fingerprint, and marker-metabolite ranges
P0	Official ConPhyMP supporting PDF	Frontiers explicitly requested ConPhyMP tables for extract/material submissions	Completed Table 1 and relevant Table 2a/chemical-profile table submitted as supplementary PDF.
P1	Full-text extraction of all primary studies	Descriptions are currently too broad for critical review	Dose, route, model, duration, controls, sample size, randomization/blinding, statistics, and safety fields for every study
P1	Independent preclinical replication	Much of the evidence is concentrated in related research groups	Replication by an independent laboratory using chemically defined material
P1	Prospective clinical trial	Current human evidence is preliminary	Registered RCT with HAMD/MADRS, sample-size calculation, allocation concealment, blinding where feasible, and adverse-event plan
P2	Safety and toxicology program	Areca, aconitum, piper, and mineral ingredients create formula-specific risks	Oral mucosa, liver/kidney, cardiovascular/cholinergic symptoms, contaminants, alkaloids, and interactions
P2	Mechanism validation	Network pharmacology and pathway associations require experimental testing	Target inhibition/knockdown, exposure confirmation, pharmacokinetics, multi-omics linked to chemistry and outcomes

## 8 Prioritized research agenda

The evidence gaps converge on a staged agenda: first define the material and its safety chemistry, then replicate preclinical effects independently, and only then test clinical efficacy prospectively.

## 9 Discussion

### 9.1 Interpretation in the wider literature

Taken together, the evidence supports a cautious interpretation. The traditional rationale for GY-13 and the preclinical evidence are sufficient to justify further investigation. They are not sufficient to claim that GY-13 is an established antidepressant treatment. The major obstacle is not merely the absence of one chromatogram; it is the absence of a reproducible chain linking authenticated and chemically defined materials to dose, exposure, pharmacological endpoint, and clinical outcome. This caution is consistent with broader depression research, in which treatment response is heterogeneous and mechanistic plausibility does not by itself predict clinical benefit (Duman and Monteggia, 2006; Pittenger and Duman, 2008; Cipriani et al., 2018). It is also consistent with the natural products literature, which emphasizes that material authentication, chemical definition, and exposure are prerequisites for reproducible pharmacology (Heinrich et al., 2020; Heinrich et al., 2022).

ConPhyMP addresses this reproducibility problem by requiring traceable materials and batch-linked chemistry (Heinrich et al., 2022). Without these data, pharmacological findings cannot confidently be generalized across GY-13 batches.

The clinical literature requires the same discipline. Adjunctive GY-13 plus sertraline may be clinically interesting, but conclusions depend on sample size, randomization, allocation concealment, blinding, baseline comparability, validated depression scales, effect sizes, adverse events, and follow-up duration. If these details are absent or inaccessible, the study should be described as preliminary, not as proof of efficacy. Future trials should be prospectively registered and should include syndrome stratification and biomarker sub-studies only as predefined secondary or exploratory analyses.

The HNS framework may still be valuable because it offers a way to translate Mongolian medicine constructs into measurable research domains without reducing them to simplistic biomarkers. Its value, however, depends entirely on prospective testing. The framework should be evaluated by measuring stress-axis load, neuroplasticity reserve, and inflammatory-oxidative burden before and after treatment with batch-defined GY-13 and by testing whether baseline profiles predict treatment response.

### 9.2 Strengths and limitations of this review

The principal strengths are an explicit evidence-tier framework, a study-selection flow diagram, record-level extraction of material and design features, separation of measured evidence from network predictions, integration of the ConPhyMP checklist, and deliberate reframing of HNS as a prospectively testable hypothesis. The review also treats absent batch and chemical data as findings that constrain reproducibility rather than filling gaps by inference.

The review also has important limitations. It was not prospectively registered; screening and extraction were not conducted by two independent reviewers; no validated design-specific risk-of-bias tool was applied; and database coverage was limited primarily to PubMed/MEDLINE, CNKI, public bibliographic records, and citation chaining. Web of Science and Scopus were not independently searched. Some Chinese studies could be evaluated only from accessible bibliographic or summary records, increasing the risk of incomplete extraction and language or availability bias. The evidence tiers are an author-defined interpretive hierarchy rather than a validated appraisal instrument. Because the evidence was sparse and heterogeneous, no meta-analysis or certainty-of- evidence grading was performed. These constraints may have led to missed records and require caution when interpreting negative searches or NR fields.

### 9.3 Limitations of the included evidence

The included preclinical literature is concentrated in a small number of related groups, with incomplete reporting of allocation, blinding, power, sample size, dose-response, pharmacokinetics, and adverse events. Model and endpoint heterogeneity prevents quantitative comparison. Network pharmacology studies add target hypotheses but cannot establish batch composition, bioavailability, or causal target engagement. The human evidence is limited to small, non-randomized or incompletely reported observations, including an adjunctive design that cannot isolate the effect of GY-13. Across designs, inadequate material definition and absent batch-linked chemistry are shared threats to reproducibility.

### 9.4 Implications and research priorities

The immediate priority is not another broad network analysis but a chemically defined, safety-qualified GY-13 preparation linked to dose and exposure. Future animal studies should use prospective protocols and design-appropriate bias controls, including randomization, allocation concealment where applicable, blinded outcome assessment, power calculations, predefined endpoints, and independent replication. Clinical studies should be prospectively registered, adequately powered, and report baseline-adjusted effects with confidence intervals, complete adverse-event categories, oral-mucosal and cardiovascular monitoring, and herb–drug interaction surveillance. HNS biomarkers should be prespecified as exploratory or secondary outcomes until independently validated.

## 10 Conclusion

GY-13 has hypothesis-generating preclinical signals relevant to depression, but current evidence does not establish clinical efficacy. Progress requires chemically defined batches, reproducible preclinical studies, and adequately powered prospective trials with standardized safety monitoring. HNS should remain a framework for generating testable associations rather than a claimed mechanism.
