# Supplementary material for: Review Areca Thirteen Pill (GY-13) for depression: a ConPhyMP-Informed critical review of material definition, pharmacological evidence, and translational Heyi-neuroplasticity-stress hypotheses
Source: Front Pharmacol. 2026 Sep 18;17:1912784. doi: 10.3389/fphar.2026.1912784 (PMC13631415; doi:10.3389/fphar.2026.1912784)
Supplement: Supplementary file 1 [file DataSheet1.pdf]

ConPhyMP Supporting Checklist for GY-13 (Areca Thirteen Pill)

This supporting file is based on a public database rerun on 3 June 2026. NR means not reported in accessible records; no missing chemical, batch, dose, or clinical numerical data were inferred.

Table 1. Materials and initial processing information extracted for GY-13 studies

ConPhyMP item	Information located in accessible records	Specific gaps / NR items	How handled in manuscript
Species and botanical drug identity	GY-13 is reported as a 13-ingredient Mongolian medicine formula. Accessible records and formula textual research list: Areca catechu L.; Aquilaria agallocha Roxb.; Myristica fragrans Houtt.; Syzygium aromaticum (L.) Merr. & L.M.Perry; Aucklandia lappa Decne.; Choerospondias axillaris (Roxb.) B.L.Burtt & A.W.Hill; Aconitum brachypodum Diels; Zingiber officinale Roscoe; Piper longum L.; Piper nigrum L.; Sal ammoniac; Angelica sinensis (Oliv.) Diels; Lepidium apetalum Willd.	Plant parts, source regions, supplier batch data, and pharmacopoeial drug names are not fully reported across all older pharmacology and clinical records.	The main manuscript lists full botanical names and treats unreported plant-part/source details as reproducibility limitations rather than inferring missing data.
Source, supplier, and batch	Cai et al. (2022) publicly reports GY-13 material purchased from Inner Mongolia Mongolian Medicine Co., Ltd. and voucher specimen No. BUCM4111403. Li et al. (2025) reports animal dose levels of 0.15, 0.3, and 0.6 g/kg in an integrated pharmacology/experimental study.	Lot number, manufacturing date, expiry date, storage conditions, complete preparation workflow, and batch-to-batch comparison are NR in most accessible records.	Reported supplier/voucher/dose information is extracted where available. NR is retained for missing manufacturing and batch metadata.
Voucher / authentication	Voucher specimen No. BUCM4111403 is reported in the accessible Cai et al. (2022) record. The record associates the material with Beijing University of Chinese Medicine herbarium/college material documentation.	Voucher numbers for most older Tong-group pharmacology studies and the Tai et al. clinical report are NR in accessible records.	Voucher information is cited only where publicly available; other studies are not treated as fully reproducible material records.
Preparation / processing	GY-13 is used as a prepared Mongolian medicine pill/tablet in the reviewed pharmacological and clinical literature. Experimental administration routes include oral/intragastric administration in rodent CUMS studies. Published records focus on pharmacological endpoints.	Detailed grinding, extraction/decoction, drying, excipient, standardization, and processing workflow are not consistently available. Processing details for Aconitum-containing and mineral-containing components are not completely traceable from the GY-13 records.	The manuscript states that the formula is historically identifiable but incompletely characterized as a reproducible test material.
Dose / route / duration in bioassays	Cai et al. (2022) uses CUMS and primary hippocampal neuron experiments; H-89 is used as a PKA inhibitor for pathway testing. Li et al. (2025) reports 0.15, 0.3, and 0.6 g/kg GY-13, administration volume 1.0 mL/100 g, and 28-day treatment. Tai et al. (2025) clinical report concerns GY-13 plus sertraline, 60 participants, 6 weeks, according to accessible bibliographic record.	Exact dose conversion, batch strength, clinical tablet/pill dose schedule, allocation details, full clinical scale values, and complete adverse-event descriptions are NR unless explicitly reported.	Dose and route are extracted when reported; the clinical report is not used for definitive efficacy claims.
Initial processing and storage	Only limited supplier/voucher information is available for selected records.	Storage temperature, humidity, light protection, transport, shelf life, and pre-test handling are NR in most records.	The manuscript identifies these as critical future reporting requirements under ConPhyMP/Four Pillars standards.
Safety-related material information	Formula contains Areca catechu and Aconitum brachypodum, and also Sal ammoniac/mineral-related material. English and Chinese safety literature indicates that areca alkaloids, processed Aconitum materials, and mineral-containing preparations require careful quality control.	GY-13 batch-specific areca alkaloid exposure, processed Aconitum toxic alkaloids, heavy metals, pesticides, and microbial limits are NR in accessible GY-13 records.	Safety is discussed separately from efficacy mechanisms; future trials must include contaminant testing and adverse-event monitoring.

Table 2a. Chemical characteristics of the GY-13 material and reporting status

ConPhyMP chemical item	Evidence located	Status / NR statement	Editorial interpretation
Chromatographic fingerprinting	No publicly reusable batch-resolved HPLC, UPLC, LC-MS, or GC-MS fingerprint for the GY-13 material reviewed in the main pharmacology and clinical records was located. Integrated pharmacology/database studies list candidate metabolites but do not provide a measured chromatogram.	NR for representative GY-13 chromatogram, annotated major peaks, retention times, relative peak areas, method parameters, and inter-batch similarity.	Database-derived candidate metabolites are not equivalent to chemical fingerprinting. This is a major reproducibility limitation.
Marker-metabolite quantification	Candidate marker classes are literature-supported: arecoline/areca alkaloids, eugenol, piperine, costunolide/dehydrocostus lactone, ligustilide-related phthalides, gingerols, quercetin, and kaempferol.	NR for batch-specific mg/g, ug/g, relative content ranges, calibration curves, replicate analyses, and acceptance limits in GY-13.	Markers are presented only as candidate quality-control targets, not as measured GY-13 contents.
Analytical validation	No accessible record reports a validated GY-13 chemical analysis method for the reviewed batch(es).	NR for reference standards, calibration range, LOD/LOQ, precision, repeatability, recovery, system suitability, and stability testing.	Future studies should provide validated HPLC/UPLC/LC-MS methods and raw chromatograms as supplementary data.
Batch comparability	No accessible record provides multi-batch chemical comparison or chromatographic similarity evaluation for GY-13.	NR for batch number, production date, expiry date, supplier batch certificate, and multi-batch fingerprint similarity.	Biological findings cannot be assumed to generalize across batches without batch-resolved QC.
Safety-relevant chemistry	Formula contains areca material and Aconitum material; adjacent literature indicates areca alkaloids and processed Aconitum alkaloids can be safety-relevant. Mineral/sal-ammoniac related quality-control issues should also be considered.	NR for arecoline/nitrosamine profiling in GY-13, processed Aconitum toxic alkaloids, heavy metals, pesticide residues, microbial limits, and contaminant certificates.	Future pharmacology and clinical trials should include safety-relevant chemistry before efficacy interpretation.
Chemical-bioactivity linkage	Network pharmacology reported 86 candidate compounds, 260 compound-related targets, and 12 MDD-shared targets; quercetin, kaempferol, and SLC6A4 were highlighted.	NR for direct correlation between measured batch metabolites and behavioral, HPA-axis, BDNF, inflammatory, or clinical endpoints.	Network predictions should be treated as hypothesis-generating until linked to measured batch chemistry and validated bioactivity.

Statement: This checklist is based on a public database rerun on 3 June 2026. No missing chemical, batch, dose, or clinical numerical data were inferred. The checklist should be read together with the main manuscript (Sections 3 and 5).
